# Inhibition of *Candida* biofilms by *Streptomyces*-derived compounds: A systematic review

**DOI:** 10.22034/cmm.2025.345437.1682

**Published:** 2026-02-01

**Authors:** Ismail Ismail, Sylvia Utami Tunjung Pratiwi, Ema Damayanti, Jaka Widada

**Affiliations:** 1 Doctoral Student in Biotechnology, Graduate School, Gadjah Mada University, Yogyakarta, Indonesia; 2 Department of Pharmacy and Pharmaceutical Technology, Faculty of Health Sciences, Almarisah Madani University, Makassar, Indonesia; 3 Department of Pharmaceutical Biology, Faculty of Pharmacy, Gadjah Mada University, Yogyakarta, Indonesia; 4 Research Center for Food Technology and Processing, National Research and Innovation Agency, Yogyakarta, Indonesia; 5 Department of Agricultural Microbiology, Faculty of Agriculture, Gadjah Mada University, Yogyakarta, Indonesia

**Keywords:** Antifungal, Antimicrobial resistance, Biofilms, *Candida*, Healthcare-associated infections, *Streptomyces*

## Abstract

**Background and Purpose:**

*Streptomyces*, a genus of Actinobacteria, is recognized for its ability to produce diverse bioactive compounds with significant antimicrobial properties. *Candida* biofilms pose a major clinical challenge due to their high resistance to antifungal agents and evasion of host immune responses. This systematic review evaluated the efficacy and mechanisms of *Streptomyces*-derived compounds in inhibiting *Candida* biofilms, with a focus on their potential clinical applications.

**Materials and Methods:**

A comprehensive literature search was performed using PubMed, Scopus, ScienceDirect, and LILACS databases, covering studies published between 2016 and 2024. The selection was limited to *in vitro* studies assessing the effects of *Streptomyces*-derived compounds on *Candida* biofilms. Articles were screened based on predefined inclusion and exclusion criteria following PRISMA guidelines. Risk of bias was evaluated using standardized tools.

**Results:**

Nine studies met the eligibility criteria. Several *Streptomyces* species were reported to produce compounds, such as pyrrolo[1,2-a]pyrazine-1,4-dione, bafilomycin derivatives, and sorbicillin, which exhibited strong antibiofilm activity. These compounds have been shown to interfere with biofilm formation through various mechanisms, including disruption of cell membranes and inhibition of hyphal adhesion and development. Notably, *Streptomyces olivaceus* and *Streptomyces chrestomyceticus* strains produced secondary metabolites that significantly reduced biofilm formation of *Candida* species.

**Conclusion:**

Compounds derived from *Streptomyces* offer promising antifungal strategies against biofilm-associated *Candida* infections. Further research is needed to explore their clinical application in combating antifungal resistance and improving treatment outcomes in healthcare settings.

## Introduction

Biofilms, particularly those formed by *Candida* species, contribute to approximately 65–80% of all hospital-acquired infections, with *Candida* biofilms specifically linked to increased mortality rates in bloodstream infections, reaching up to 49% in some cohorts [1, 2]. Their inherent resistance to antifungal agents and evasion of host immune responses significantly compromise treatment efficacy and clinical outcomes [3]. These structured communities of microorganisms, encased in a self-produced extracellular matrix (ECM), exhibit enhanced resistance to antimicrobial agents and host immune responses [4]. This resistance is attributed to several key features of the ECM, including the physical barrier that impedes drug penetration, sequestration of antifungal agents, and the upregulation of resistance-related genes, such as efflux pumps and stress-response regulators [5, 6].

*Candida* biofilms are especially problematic as they frequently colonize medical devices, such as catheters, implants, and prosthetic joints. It is estimated that up to 70% of catheter-related bloodstream infections involve *Candida biofilms*, with associated mortality rates ranging from 30% to 49% [7–10]. These infections can lead to increased hospital stays and treatment costs exceeding $34,000 per patient in the United States alone [11]. Among *Candida* species, *Candida albicans*, *Candida tropicalis*, *Candida parapsilosis*, and *Candida glabrata* are well-documented for their robust biofilm-forming capabilities on medical devices [7, 12, 13]. These biofilms serve as persistent reservoirs of infection and have been directly implicated in invasive candida infection and catheter-related bloodstream infections, particularly in immunocompromised patients [7, 14]. Furthermore, the presence of *Candida* biofilms has been linked to significantly higher mortality rates in patients with candidemia, with studies reporting mortality as high as 49% in biofilm-positive cases, compared to 21% in biofilm-negative cases. This underscores the critical need for effective strategies to prevent and manage biofilm-associated infections in clinical settings [15, 16]. As biofilms are estimated to be involved in over 80% of all microbial infections, understanding their formation, characteristics, and impact on healthcare is crucial for developing targeted therapies and improving patient care [17, 18].

Biofilm-associated infections present significant challenges in clinical settings due to their inherent resistance to conventional antimicrobial treatments and the host immune system, necessitating the development of effective antifungal strategies. These infections are particularly difficult to treat since the biofilm matrix acts as a physical barrier that limits the penetration of antifungal agents, while the microorganisms within the biofilm exhibit altered metabolic states and enhanced resistance mechanisms, such as efflux pumps and enzyme production, which further complicate treatment efforts [19, 20]. This resistance not only leads to persistent and recurrent infections but also contributes to the global issue of antimicrobial resistance, making it increasingly difficult to manage and eradicate these infections with existing therapies [19, 21, 22]. The need for effective antifungal strategies is further underscored by the fact that biofilm-associated infections are often linked to medical devices, such as catheters, prosthetic joints, and implants, which serve as surfaces for biofilm formation and reservoirs for continuous infection [23, 24].

Consequently, these infections are associated with prolonged hospital stays, increased healthcare costs, and higher morbidity and mortality rates [20]. Therefore, the development of novel antifungal strategies is urgently needed, particularly agents that target biofilm-specific pathways, such as hyphal formation, ECM biosynthesis, and efflux pump regulation [5, 25, 26]. Current antifungals, including azoles and echinocandins, exhibit limited efficacy against mature *Candida* biofilms, with resistance levels reported to be up to 1,000-fold higher, compared to their planktonic counterparts [27–29].

Promising alternatives include natural products derived from *Streptomyces* sp., quorum-sensing inhibitors, and enzymes that degrade biofilm matrices, which offer distinct mechanisms to circumvent antifungal resistance [30, 31]. Given their structural diversity and multifaceted modes of action, *Streptomyces*-derived metabolites represent a particularly compelling frontier in antifungal drug development with the potential to reduce the burden of biofilm-associated infections and address the escalating challenge of antifungal resistance in healthcare [19, 21, 22].

Primary objective of this systematic review was to comprehensively identify and synthesize all relevant studies investigating the use of *Streptomyces*-derived compounds in inhibiting *Candida* biofilms. By conducting an exhaustive search of the scientific literature, this review aimed to collate and analyze the current body of knowledge regarding the efficacy, mechanisms of action, and potential applications of *Streptomyces* metabolites in combating biofilm-associated *Candida* infections. Through this synthesis, the present review sought to elucidate the most promising compounds, their specific effects on *Candida* biofilm formation and maintenance, and their potential advantages over existing antifungal treatments. Additionally, this review aimed to identify gaps in the current research, highlight areas for future investigation, and provide a comprehensive resource for researchers and clinicians working towards developing novel strategies to address the challenges posed by *Candida* biofilms in healthcare settings.

## Materials and Methods

### Databases searched

The systematic review was conducted in strict adherence to the Preferred Reporting Items for Systematic Reviews and Meta-Analyses (PRISMA) 2020 guidelines [32, 33]. Although the review protocol was not registered in PROSPERO or any other public registry, the systematic review was conducted rigorously to ensure methodological transparency and reproducibility. Several online databases were employed to conduct this study, including PubMed/MEDLINE, Scopus, ScienceDirect, and LILACS, with access on 4 July 2024.

### Keywords and search terms

The search strategy utilized a combination of Medical Subject Headings terms and free-text keywords, including:

- *Streptomyces* OR Actinobacteria
- *Candida* OR yeast OR fungal
- Biofilm OR "microbial communities"
- Inhibit* OR disrupt* OR prevent*
- Antifungal OR antimicrobial OR anti-infective
- Secondary metabolite* OR compound* OR molecule*

These terms were combined using Boolean operators (AND, OR) to create comprehensive search strings tailored to each database. Table 1 summarizes the search terms used across databases to identify studies evaluating the role of *Streptomyces*-derived compounds in inhibiting *Candida* biofilms. Duplicate records were identified and removed using Zotero and Rayyan prior to the screening process [34].

**Table 1:** Keywords for searching method

No.	Sources	Keyword
1.	PubMed	*Streptomyces*' AND anti biofilm AND *Candida*'
2.	Scopus	Title-ABS-Key (*Streptomyces*' AND anti AND biofilm AND '*Candida*')
3.	Science Direct	*Streptomyces*' AND anti biofilm AND '*Candida*'
4.	LILACS	Title-ABS-Subject *Streptomyces*' AND anti biofilm AND '*Candida*'

### Eligibility and inclusion/exclusion criteria

The inclusion criteria for this systematic review were as follows: (1) original research articles published in peer-reviewed journals; (2) studies focused on *Streptomyces*-derived compounds and their effects on *Candida* biofilms; (3) *in vitro* studies; (4) articles published in English; and (5) studies published within the last 10 years (2016–2024) to ensure relevance. Studies were excluded if they met any of the following exclusion criteria: (1) review articles, conference abstracts, and book chapters; (2) studies not specifically addressing *Candida* biofilms; (3) research focused solely on planktonic *Candida* cells; (4) studies using compounds not derived from *Streptomyces*; and (5) articles not available in full text.

### Study selection

The search results were initially screened by title and abstract to remove irrelevant studies. The remaining articles were then subjected to full-text review to determine their eligibility based on the inclusion and exclusion criteria. Reference lists of included studies and relevant review articles were also manually searched to identify additional eligible studies. This comprehensive search strategy aimed to capture all relevant literature on the topic while maintaining a focus on high-quality, peer-reviewed research specifically addressing the role of *Streptomyces* compounds in inhibiting *Candida* biofilms.

### Data collection process

The data collection process involved systematic extraction of relevant information from each eligible article. A standardized data extraction form was developed to capture key variables, including author(s) and year, study title, *Streptomyces* species, *Candida* species, identified compounds, and antifungal activity (e.g., minimum inhibitory concentration [MIC], reported in µg/mL). Data extraction was performed independently by one reviewer (Author A) and verified by a second reviewer (Author B) to ensure accuracy and consistency. Any discrepancies were resolved through discussion and consensus. In cases where essential data were missing or unclear, efforts were made to contact the corresponding authors. If no response was received, missing data were documented as “not reported” and excluded from quantitative synthesis. The entire process was guided by PRISMA recommendations to maintain methodological rigor and data integrity.

### Data extraction and organization

A pre-designed data extraction form was used by one researcher (A), with the aim of answering the research questions guiding this systematic review, and was subsequently verified by a second researcher (B). In case of disagreement, consensus was reached through discussion and based on factual evidence. The PRISMA statement (Page et al., 2021) was referred for data organization and evaluation, and Zotero and Rayyan software programs, or spreadsheets, were used to facilitate the data extraction and organization [32].

### Assessment of risk of bias

Risk of bias (ROB) was assessed independently by three reviewers using a customized checklist based on established criteria for *in vitro* studies, adapted from SYRCLE and ToxRTool frameworks [33–36]. Fourteen predefined parameters were used to evaluate methodological quality, including clarity of objectives, description of microorganisms, study design, MIC determination, biofilm inhibition testing, replication, controls, statistical analysis, and reporting transparency.

Each criterion was scored “Y” (yes, adequately reported) or “N” (no or unclear). Each “Y” was assigned a value of 1 point. Total scores were categorized as follows: 0–5 = high ROB (+++), 6–7 = medium ROB (++), and 8–10 = low ROB (+). Discrepancies among reviewers were resolved by discussion to reach consensus.

To visualize the risk of bias across studies and domains, we used the robvis tool (https://www.riskofbias.info/welcome/robvis-visualization-tool#h.p_uR6_sUdFGIHy), which generated both traffic-light plots and summary bar charts. Inter-rater reliability was evaluated using Fleiss’ Kappa, yielding a coefficient of 0.79, indicating substantial agreement. Full ROB scoring is provided in Supplementary Table S1.

### Statistical analysis

Data were compiled and organized using Microsoft Excel and GraphPad version 9.3.1 was used for data analysis and graphical visualization. For continuous variables, such as MIC values, descriptive statistics, including mean, standard deviation, and range, were calculated. Frequencies and percentages were used to summarize categorical variables, such as the distribution of *Candida* species or compound types. Comparative plots (bar charts and scatter plots) were generated to visualize the relative efficacy of *Streptomyces*-derived compounds across different *Candida* strains. No inferential statistical tests (e.g., analysis of variance or t-tests) were applied due to heterogeneity in study design, outcome measures, and lack of standardized control groups across the included studies.

## Results

### Study selection

This systematic review included articles published between 2016 and 2024 to ensure the inclusion of recent and relevant findings. A total of 628 records were identified from electronic databases, namely PubMed (n=13), Scopus (n=27), ScienceDirect (n=570), and LILACS (n=18). After removal of 25 duplicates, 603 records remained for title and abstract screening. During screening, 489 records were excluded based on type of publication, including review articles (n=262), encyclopedias (n=21), book chapters (n=150), conference abstracts (n=1), mini reviews (n=4), and other irrelevant formats (n=51). A total of 114 full-text articles were then assessed for eligibility using the PICOS (population, intervention, comparison, outcomes, study design) framework and predefined inclusion/exclusion criteria. Of these, 112 articles were excluded due to irrelevant outcomes (n=41), inappropriate study designs (n=33), or non-target populations (n=38) (Supplementary Table S2). An additional seven articles were identified through citation searching, all of which met the eligibility criteria and were included in the review. Therefore, a total of nine articles were included in the review. However, one article was subsequently excluded during the risk of bias assessment due to insufficient methodological data, resulting in eight final studies. The study selection process is illustrated in the PRISMA 2020 flowchart (Figure 1), in accordance with the PRISMA guidelines [32, 33].

**Figure 1:**
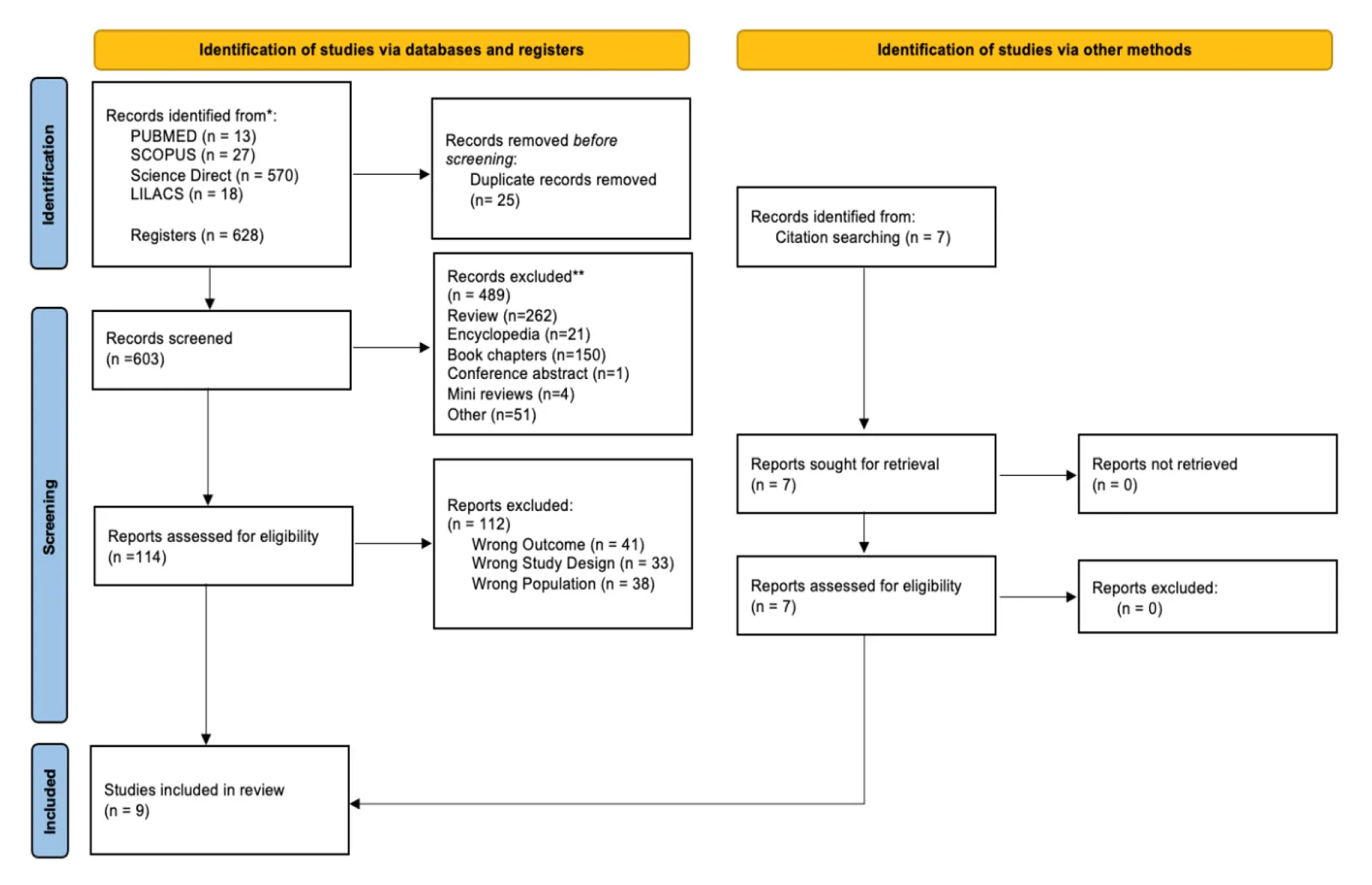
Search strategy-preferred reporting items for systematic reviews and meta-analyses flow diagram

### Risk of bias assessment

Before data collection and statistical analyses, the nine studies selected in this systematic review were evaluated for quality using a ROB evaluation. The evaluation considered whether the articles provided information on various criteria, including the source of microorganisms, the authenticity of *Streptomyces* sp. strains, clearly stated objectives, determination of MIC, biofilm inhibition assay, replicate analyses, use of positive controls, identification of specific compounds and explanation of the mechanism of action, microorganism test (*Candida* sp.), which were categorized as high (+++), medium (++), or low (+) ROB. Each quality assessment criterion was represented by a different colored box, with the authors assigning a score to each parameter. The average total score was rounded to the nearest whole number, and the ROB of the selected study was calculated as follows:

- Reporting 0-5 items = high ROB (score +++)
- Reporting 6-7 items = medium ROB (score ++)
- Reporting 8-10 items = low ROB (score +)

All three authors made these judgments at the outcome level (Figure 2). Of the nine studies, one had a high ROB (score +++), two studies had a medium ROB (score ++), and six studies had a low ROB (score +). If the mean value for a parameter fell between classes, the mean value was rounded to the nearest whole number. One study [39] scored five (high ROB) and was excluded from further analysis. The remaining seven studies scored between 7 and 10 (out of 10) and were used for data synthesis and conclusion formulation.

**Figure 2:**
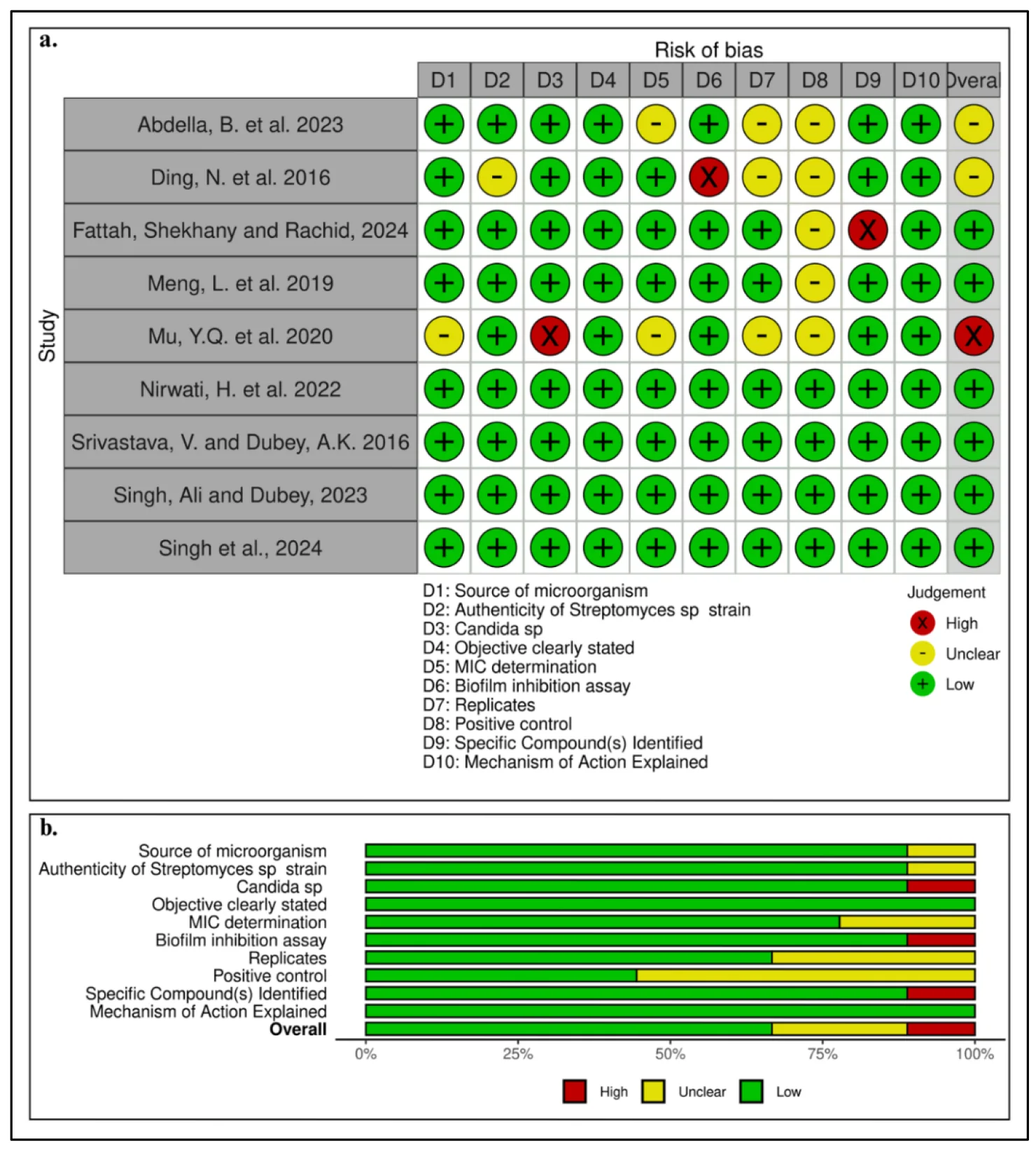
Risk of bias assessment of the included *in vitro* studies using the robvis tool. (a) Traffic light plot showing domain-level judgment for each study across 10 predefined methodological criteria, namely D1: source of microorganism; D2: authenticity of *Streptomyces* sp. strain; D3: *Candida* species identification; D4: objective clearly stated; D5: determination of minimum inhibitory concentration; D6: biofilm inhibition assay; D7: replicates; D8: positive control; D9: compound identification; and D10: mechanism of action explained. Each criterion is rated as low risk (green “+”), unclear (yellow “–”), or high risk (red “×”) of bias; (b) Summary bar chart illustrates the overall distribution of risk of bias judgments across all studies per domain. Most domains were assessed as low risk; however, positive control use and replicates had a higher proportion of high or unclear risk.

A risk assessment of the various research parameters related to *Streptomyces* sp. compounds as biofilm inhibitors for *Candida* sp. was conducted using the robvis tool (https://www.riskofbias.info/welcome/robvis-visualization-tool#h.p_uR6_sUdFGIHy) to ensure the validity and reliability of the study. This tool assesses the level of risk of bias for each parameter using a color-coded system.

The graph above assesses several essential aspects of the study using color coding to indicate the level of risk of bias of each parameter, showing that the source of microorganisms, MIC determination, biofilm inhibition assay, and clearly described objectives had a low risk of bias. It also indicates that the authenticity of *Streptomyces* sp. strains, replicates, positive controls, and the described mechanism of action had a low to moderate risk of bias. Moreover, it demonstrates that the use of *Candida* sp. and identification of specific compounds had a low risk of bias, but with some high risk, providing an overall assessment with a majority of low risk of bias.

### Study characteristics and variations

This review aimed to systematically collect information about Molecular Compounds from *Streptomyces* sp. that act as biofilm inhibitors in *Candida* sp. The review focused on *Streptomyces*-derived compounds and their impact on *Candida* biofilms, examining studies published in the last 9 years (2016-2024). Among the final eight records included in the review, the presence of compounds produced by *Streptomyces* was characterized by the evaluation of the inhibitory effect of secondary metabolites produced by *Streptomyces* sp. on biofilm-related *Candida* sp. The evaluation considered *Streptomyces* strains, secondary metabolites produced, *Candida* strains used as test microorganisms, biofilm testing methods, experimental conditions, controls used in testing, and statistical analysis employed. The review also considered the variation of analysis, such as metabolite extraction methods, *Candida* biofilm testing methods, quantification techniques, compound concentrations, combination therapy, molecular-based analysis, and time-kill kinetic testing.

A review of the eight records showed heterogeneity in the characteristics analyzed and reported the potential of molecular compounds from *Streptomyces* for *Candida* biofilm inhibition. The overall characteristics and variations of the analysis are illustrated in Figure 3.

**Figure 3:**
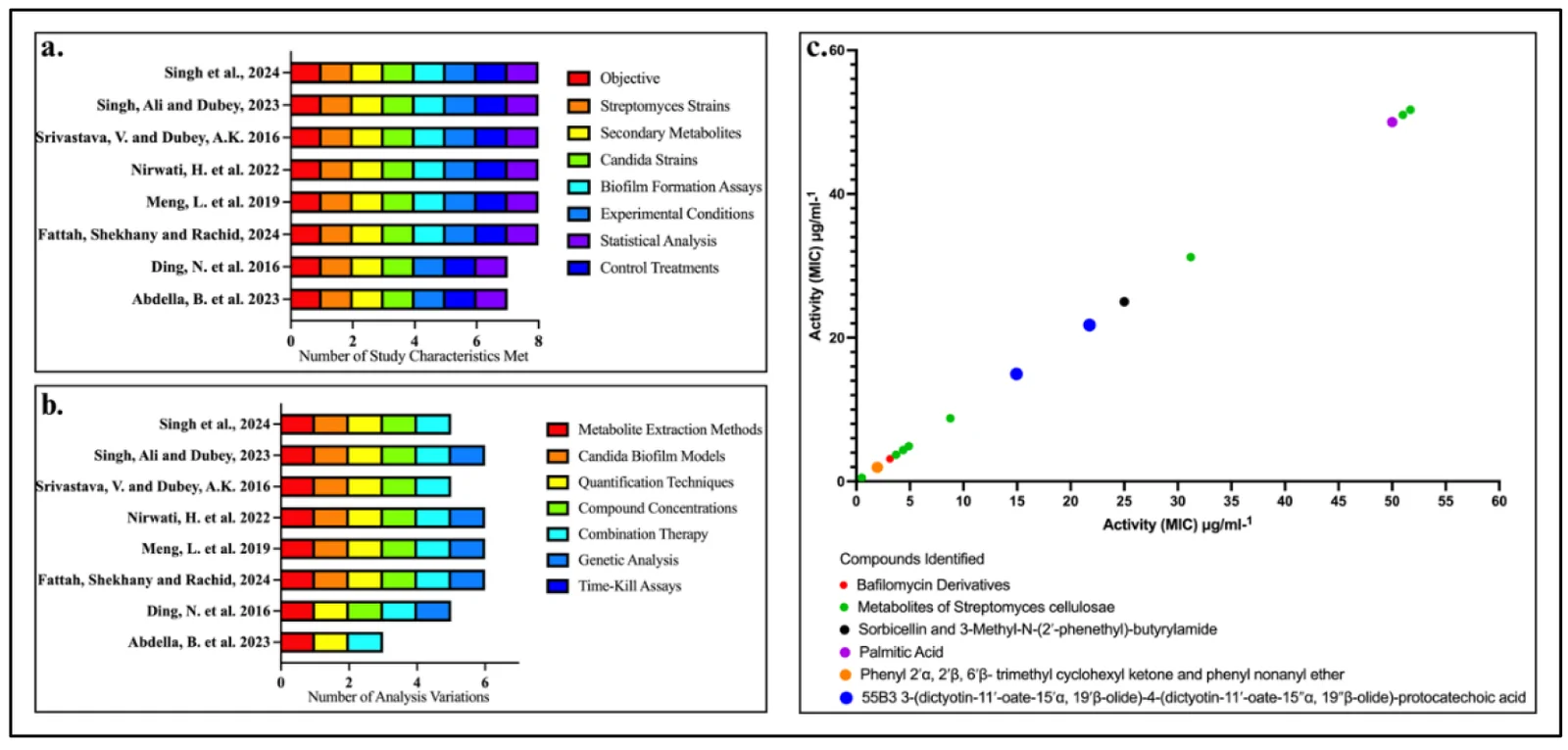
Overview of study characteristics and variation among the included articles. (A) general study attributes, such as year of publication, geographic origin, and strain identification of *Streptomyces* sp.; (B) experimental methodologies applied across studies, including minimum inhibitory concentration (MIC) testing, biofilm inhibition assays, and identification of active compounds; and (C) reported effects of *Streptomyces*-derived metabolites on *Candida* sp. biofilms, including MIC values. This figure highlights methodological diversity and variability in compound efficacy across the selected studies.

### Streptomyces compounds and their effects on Candida sp.

Based on the reviewed studies, here are the key findings regarding *Streptomyces* compounds and their effects on *Candida* sp. are summarized in Table 2.

**Table 2:** *Streptomyces* compounds and their effects on *Candida* sp.

Source of microorganism	*Streptomyces* species	Compounds identified	Effects on *Candida* sp.	Testing method	References
Soft coral *Sarcophyton glaucum*	*Streptomyces* sp. Strain HC14	Pyrrolo[1,2-a]pyrazine-1,4-dione, hexahydro-3-(phenylmethyl)-3 and di-n-octyl	*Candida albicans*	*In vitro*	[37, 38]
*Elephas maximus* feces	*Streptomyces albolongus*	Bafilomycins, 19-methoxybafilomycin C1 amide	*Candida parapsilosis*	*In vitro*	[39]
		21-deoxybafilomycin A1			
		21-deoxybafilomycin A2			
		Sesquiterpenoid			
		(1b, 4b, 4a, 8aa) -4,8a-dimethyloctahydronaphthalene-1,4a (2H) -diol			
Soil	*Streptomyces cellulosae*	Metabolites of *Streptomyces cellulosae*	*Candida albicans*	*In vitro*	[40]
Sediment	*Streptomyces olivaceus* SCSIO T05	Sorbicillin	*Candida albicans*	*In vitro*	[41]
		3-methyl-N-(2'-phenethyl)-butyrylamide			
Cajuput rhizosphere soil	*Streptomyces* sp. GMR22	Palmitic acid	*Candida albicans*	*In vitro*	[42]
Soil	*Streptomyces chrestomyceticus* strain ADP4	Metabolites of *Streptomyces chrestomyceticus* strain ADP4	*C. albicans* ATCC 10231, *C. albicans* ATCC 90028, *C. albicans* ATCC 36082, *C. albicans* ATCC Y0119, *C. albicans* ATCC 1239, *C.krusei* ATCC 6258, *C. krusei* ATCC 766.1, *C.tropicalis* ATCC 750 and *C.parapsilosis* ATCC 22019	*In vitro*	[43]
Soil	*Streptomyces chrestomyceticus* strain ADP4	Phenyl 2′α, 2′β, 6′β- trimethyl cyclohexyl ketone Phenyl nonanyl ether	*C.albicans* ATCC 10231, *C.krusei* ATCC 6258, *C.tropicalis* ATCC 750, *C.parapsilosis* ATCC 90018, and *C.auris* CBS 12372	*In vitro*	[44]
Soil	*Streptomyces chrestomyceticus* strain ADP4	55B3 3-(dictyotin-11′-oate-15′α, 19′β-olide)-4-(dictyotin-11′-oate-15″α, 19″β-olide)-protocatechoic acid	*C.albicans* ATCC 10231 *C.auris* CBS 12372	*In vitro*	[45]

### Efficacy and mechanisms of action of streptomyces compounds

This systematic review identified several *Streptomyces*-derived compounds that demonstrated significant biofilm inhibition against *Candida* sp. The key compounds and their mechanisms of action are discussed below:

### 1. Pyrrolo [1,2-a] pyrazine-1,4-dione, hexahydro-3-(phenylmethyl)-3, and di-n-octyl

Source of the compounds is *Streptomyces* sp. HC14. Mechanism of action of these compounds, identified through gas chromatography-mass spectrometry, demonstrates high potential for anti-*Candida* activity by specifically targeting the cytochrome monooxygenase (CYP51) enzyme. This enzyme is essential for ergosterol synthesis in *Candida albicans*, thereby inhibiting the growth and survival of the pathogen [41, 49]. The chemical structures of pyrrolo[1,2-a]pyrazine-1,4-dione, hexahydro-3-(phenylmethyl)-3 and di-noctyl phthalate are shown in Figure 4a and 4b, respectively.

**Figure 4:**
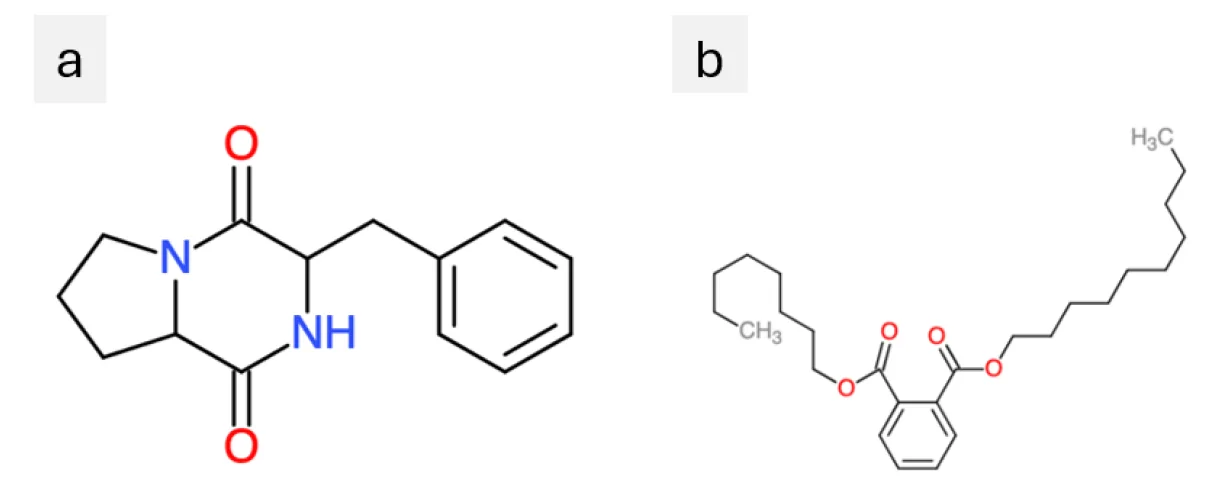
Pyrrolo [1,2-a] pyrazine-1,4-dione, hexahydro-3-(phenylmethyl)-3 (a); Di-n-octyl phthalate (b).

### 2. Bafilomycin derivatives

Sources of the compounds include *Streptomyces halstedii* and other *Streptomyces* strains. Their mechanism of action involves bafilomycin derivatives, such as bafilomycin B1 and C1, which inhibit the growth of *Candida parapsilosis* biofilms. These compounds exert their effects by disrupting the vacuolar H+-ATPase, an enzyme crucial for maintaining intracellular pH and ion homeostasis. This disruption impairs cellular functions, ultimately leading to biofilm inhibition [39, 46, 47]. The structures of bafilomycin B1 and C1 are presented in Figure 5a,b

**Figure 5:**
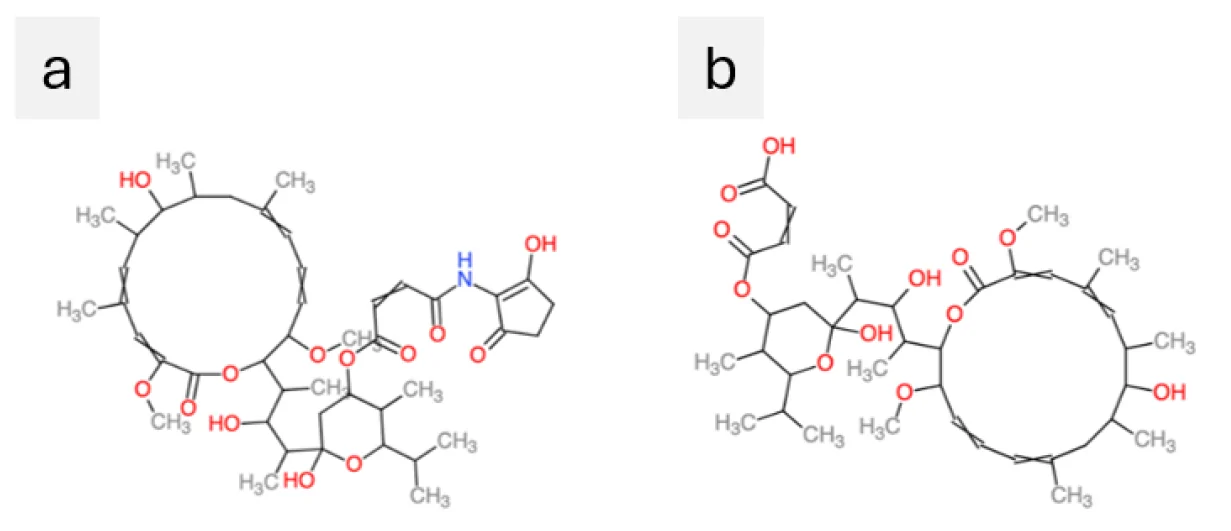
Bafilomycin B1 (a); Bafilomycin C1(b)

### 3. Sorbicellin and 3-methyl-N-(2′-phenethyl)-butyrylamide

Source of the compounds is *Streptomyces olivaceus* SCSIO T05. Their mechanism of action includes the inhibition of hyphal and biofilm formation of *Candida albicans*. Notably, 3-methyl-N-(2′-phenethyl)-butyrylamide demonstrated effectiveness in inhibiting *Candida* infection in mouse oral mucosal models by regulating the expression of genes associated with filament formation and cell adhesion [41, 48] (Figure 6).

**Figure 6:**
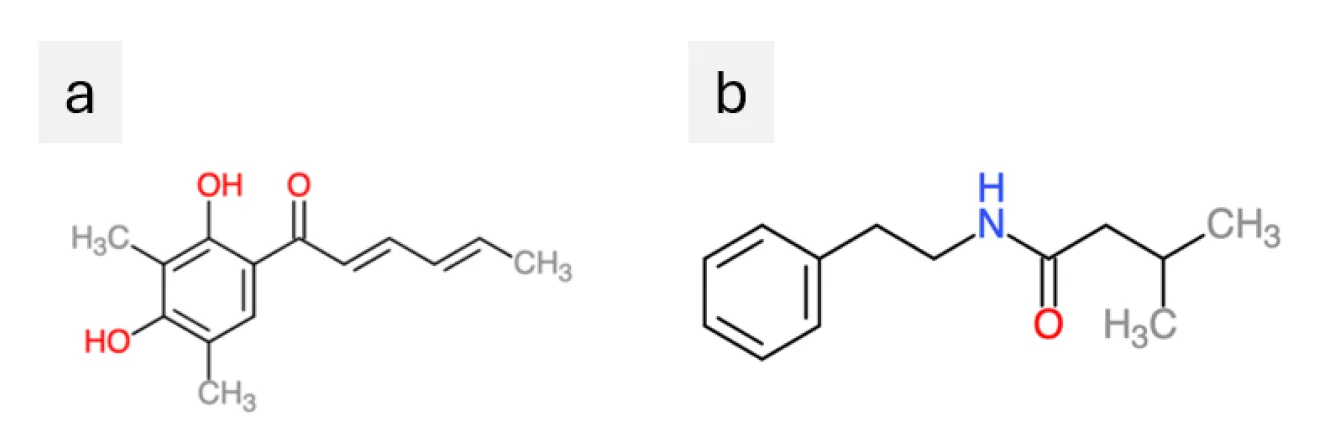
Sorbicellin (a); 3-Methyl-N-(2′-phenethyl)-butyrylamide (b)

### 4. Palmitic Acid

*Streptomyces* sp. GMR22 produces palmitic acid as the main component of the chloroform extract, which has antifungal and antibiofilm properties by the mechanism of disrupting cell membrane integrity and inhibiting *Candida* cell adhesion on the surface, which is supported by the presence of a gene cluster encoding fatty acid biosynthesis enzymes [42]. The chemical structure of palmitic acid is shown in Figure 7.

**Figure 7:**
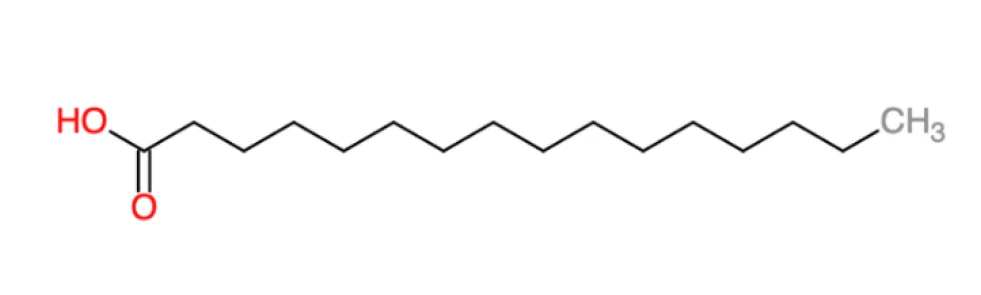
Palmitic acid

### 5. Phenyl 2′α, 2′β, 6′β- trimethyl cyclohexyl ketone and Phenyl nonanyl ether

*Streptomyces chrestomyceticus* strain ADP4 produces metabolites that exhibit potent anti-*Candida* activity by disrupting cell membranes, causing leakage of cell contents, as well as by inhibiting adhesion to polystyrene surfaces and preventing hyphae formation, which is an important step in biofilm development [43-45] (Figure 8).

**Figure 8:**
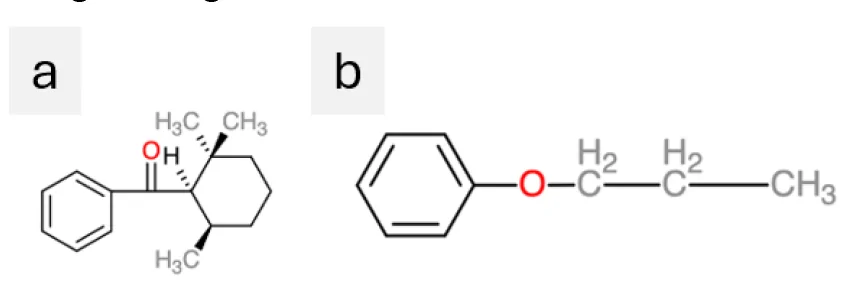
Phenyl 2′α, 2′β, 6′β- trimethyl cyclohexyl ketone (a); Phenyl nonanyl ether (b)

### 6. Actinomycin D

*Streptomyces parvulus* and *Streptomyces luteus* produce actinomycin D, which inhibits biofilm formation by downregulating the ica locus responsible for the production of polysaccharide intercellular adhesins. This decrease in polysaccharide intercellular adhesins production reduces cell surface hydrophobicity, thereby preventing cell adhesion and aggregation necessary for biofilm formation. In addition, actinomycin D disrupts the biofilm matrix and inhibits cell adhesion to glass-like surfaces [51, 52] (Figure 9).

**Figure 9:**
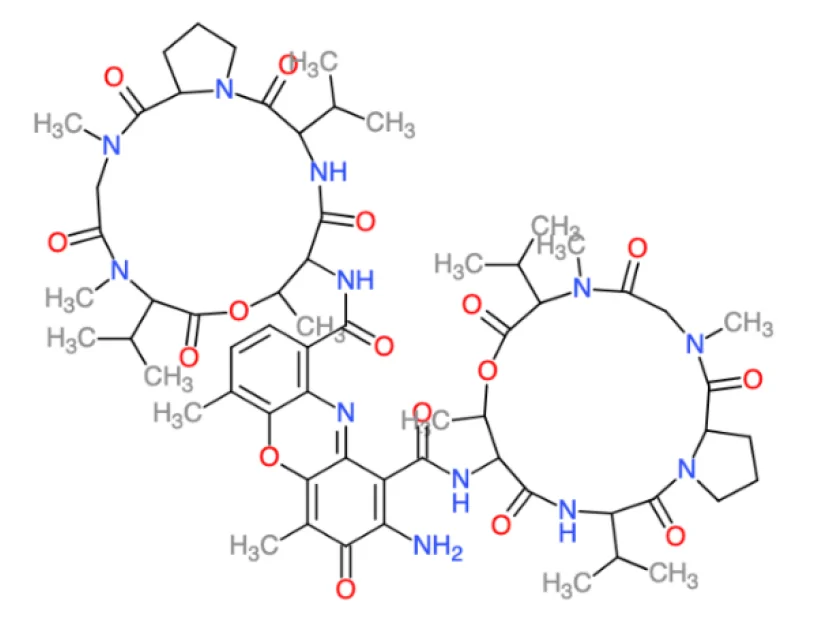
Actinomycin D

### 7. Filipin-like compounds

Various *Streptomyces* species produce filipin-like compounds that exhibit superior inhibition against *Candida* biofilms, compared to amphotericin B. These compounds disrupt the biofilm matrix and inhibit the growth of *Candida glabrata* and *Candida krusei* through a mechanism involving binding to ergosterol in the fungal cell membrane, leading to increased membrane permeability and cell death [30].

This article highlighted the significant potential of compounds derived from *Streptomyces* to prevent *Candida* biofilms. Various compounds, including actinomycin D, palmitic acid, filipin-like compounds, bafilomycin derivatives, and 3-methyl-N-(2′-phenethyl)-butylamide, have demonstrated potent anti-biofilm activity through different mechanisms, such as disruption of the biofilm matrix, inhibition of cell adhesion, and interference with crucial cellular processes. Table 3 provides details of different studies that examined the antibiofilm activity of compounds produced by *Streptomyces* species against *Candida* sp. Each row in the table represents specific study characteristics, including the *Streptomyces* species, solvent used for extraction, identified compounds, effects against *Candida* sp., type of biofilm assay, and antifungal activity measured by MIC in μg/ml.

**Table 3:** This table illustrates the variation in *Streptomyces* species, extraction solvents, compounds produced, and the effectiveness of these compounds in inhibiting *Candida* sp. biofilm formation using various test methods

Study	Streptomyces species	Solvent used in extraction	Compounds identified	Effects on Candida sp.	Biofilm formation assays	Activity (MIC) μg/ml^-1^
Ding, N. et al. 2016	*Streptomyces albolongus*	Petroleum Ether- Ethyl acetate	Bafilomycin derivatives	*C. parapsilosis*	MTT assay	3.13
Fattah, Shekhany, and Rachid, 2024	*Streptomyces cellulosae*	Ethyl acetate	Metabolites of *Streptomyces cellulosae*	*C. albicans*	Crystal Violet Assay	0.5
Meng, L. et al. 2019	*Streptomyces olivaceus* SCSIO T05	Aseton dan Butanon	Sorbicellin and 3-Methyl-N-(2′-phenethyl)-butyrylamide	*C. albicans* SC5314	Crystal Violet Assay	25
Nirwati, H. et al. 2022	*Streptomyces* sp. GMR22	*n*-hexane and chloroform	Palmitic Acid	*C. albicans* ATCC 10231	MTT assay.	50
Srivastava, V. and Dubey, A.K. 2016	*Streptomyces chrestomyceticus* strain ADP4	Ethyl acetate	Metabolites of *Streptomyces cellulosae*	*C. albicans* ATCC 10231	MTT assay	8.77 (±3.3)
				*C. albicans* ATCC 90028		4.36 (±1.6)
				*C. albicans* ATCC 36082		3.70 (±1.8)
				*C. albicans* ATCC Y0119		4.90 (±1.2)
				*C. albicans* ATCC 1239		4.36 (±1.6)
				*C. krusei* ATCC 6258		31.20
				*C. krusei* ATCC 766.1		51.70 (±10)
				*C. tropicalis* ATCC 750		51.0 (±10.2)
				*C. parapsilosis* ATCC 22019		51.70 (±10)
Singh, Ali, and Dubey, 2023	*Streptomyces chrestomyceticus* strain ADP4	Ethyl acetate	Phenyl 2′α, 2′β, 6′β- trimethyl cyclohexyl ketone and phenyl nonanyl ether	*C. albicans* ATCC 10231,	INT assay	1.95
Singh et al., 2024	*Streptomyces chrestomyceticus* strain ADP4	Ethyl acetate	55B3 3-(dictyotin-11′-oate-15′α, 19′β-olide)-4-(dictyotin-11′-oate-15″α, 19″β-olide)-protocatechoic acid	*C. albicans* ATCC 10231,	INT assay	14.94±0.17
				*C. auris* CBS 12372	INT assay	21.75±1. 5

MIC: minimum inhibitory concentration, MTT assay: 3-(4,5-dimethylthiazol-2-yl)-2,5-diphenyltetrazolium bromide, INT assay: iodonitrotetrazolium or 2-(4-iodophenyl)-3-(4-nitrophenyl)-5-phenyl-2H-tetrazolium

## Discussion

*Candida* biofilm-related infections have become a serious global health concern, particularly due to the rising incidence of antifungal resistance among high-risk strains, such as *Candida albicans* and *Candida auris*. These species are frequently implicated in device-associated infections and bloodstream infections in ICU patients, where biofilm formation on catheters, prosthetic devices, and implants leads to chronic and hard-to-treat infections. Notably, *C. auris* biofilms have demonstrated up to 1,000-fold increased resistance to azoles and echinocandins, compared to their planktonic counterparts [49, 50]. This resistance contributes significantly to elevated morbidity, mortality, and healthcare costs, particularly in immunocompromised and critically ill populations. The urgent need for new therapeutic approaches is underscored by the failure of conventional antifungals to eradicate mature biofilms. In this context, *Streptomyces* spp. represent a promising source of bioactive metabolites with antibiofilm properties, and merit further investigation for their potential role in combating resistant *Candida* biofilms [30].

Several studies included in this review reported MIC values of *Streptomyces*-derived compounds that were comparable or even superior to those of conventional antifungals. For example, pyrrolo[1,2-a]pyrazine-1,4-dione, hexahydro-3-(phenylmethyl)-3 demonstrated MIC values as low as 0.78 μg/mL against *Candida albicans*, which is within or lower than the MIC range typically reported for fluconazole against susceptible strains [51]. Moreover, some compounds exhibited antibiofilm activity against fluconazole-resistant strains, a major clinical challenge [43, 44]. While amphotericin B remains effective against most *Candida* biofilms, its toxicity profile limits its clinical use. In contrast, the *Streptomyces*-derived compounds identified in this review demonstrated high *in vitro* potency with potentially lower toxicity; however, *in vivo* data are still lacking. These findings support the clinical potential of these compounds, particularly for use against drug-resistant biofilm-forming *Candida* species. However, direct comparative studies and *in vivo* assessments are essential to confirm their therapeutic potential.

One of the key compounds highlighted in this review, *pyrrolo[1,2-a]pyrazine-1,4-dione, hexahydro-3-(phenylmethyl)-3*, may exert its antifungal effect by targeting cytochrome P450 14α-demethylase (CYP51), thereby interfering with ergosterol biosynthesis in *Candida albicans* [38]. While direct experimental validation is pending, molecular docking studies support this hypothesis. Its recurring identification across studies—ranging from *Streptomyces* isolates active against *C. albicans* [51], *Pyricularia oryzae* [52], and root-nodule bacteria [53] suggests a broad antimicrobial mechanism, potentially involving disruption of fungal membrane integrity or metabolic pathways. Given its low MIC and multi-target potential, this compound stands out as a candidate for further investigation in antifungal drug development [38, 51].

The provided context does not include specific information on the mechanism of action of bafilomycin derivatives against *Candida albicans*. Bafilomycin is a known inhibitor of the vacuolar-type H+-ATPase (V-ATPase), but without direct evidence or studies presented in the context regarding the anti-*Candida* activity of bafilomycin derivatives, a detailed mechanism cannot be ascertained. However, it is noteworthy that several studies have investigated the antifungal mechanisms of various compounds. For instance, α-pinene was shown to interact with ergosterol in the cellular membrane and inhibit biofilm formation [54]. Antifungal mechanism of geraniol did not involve the cell wall or ergosterol binding but inhibited pseudohyphae and chlamydoconidia formation [55]. Moreover, chitosan hydrogel was found to induce primary lesions of the cytoplasmic membrane [56]. A review highlighted antifungal mechanisms of natural products, including membrane-active effects and enzyme inhibition [31]. Cationic peptides disrupted cell wall structures and compromised cell membrane permeability [57]. Ionic liquids affected cell volume, membrane permeability, and ergosterol content [58].

These findings suggest that antifungal agents can have diverse mechanisms of action against *Candida* sp. In summary, while the mechanism of action of bafilomycin derivatives against *C. albicans* is not provided in the context, the literature indicates various mechanisms by which different antifungal agents exert their effects. These include interactions with the cell membrane, inhibition of cell wall synthesis, and disruption of cellular processes. Further research would be required to elucidate the specific mechanisms of bafilomycin derivatives in the context of anti-*Candida* activity.

Sorbicillin and 3-methyl-N-(2′-phenethyl)-butyrylamide have been identified as compounds with potent anti-*Candida* activity. Specifically, sorbicillin and 3-methyl-N-(2′-phenethyl)-butyrylamide were found to exhibit strong activity against the morphological transition, adhesion activity, cytotoxicity, and adhesion to human cells of *Candida albicans* in a dose-dependent manner. In particular, 3-methyl-N-(2′-phenethyl)-butyrylamide was shown to inhibit *C. albicans* infection in mouse oral mucosal models. Mechanism of action for these compounds appears to involve the regulation of gene expression levels of HWP1, TEC1, ALS1, IFD6, and CSH1, which are associated with filament formation and cell adhesion [41]. It is noteworthy that while these compounds have demonstrated efficacy against *C. albicans* pathogenicity, there is no direct mention of sorbicillin or 3-methyl-N-(2′-phenethyl)-butyrylamide in the other papers provided. These mechanisms may offer insights into the broader context of antifungal strategies, although they are not directly related to the compounds in question. In summary, sorbicillin and 3-methyl-N-(2′-phenethyl)-butyrylamide have been shown to be effective against *C. albicans* by regulating the expression of key genes involved in morphogenesis and adhesion, which are critical for the pathogenicity of this fungus [41]. While the specific mechanisms of action for these compounds are not detailed in the other papers, the general strategies employed by various antifungal agents provide a backdrop for understanding the multifaceted approaches to combating *Candida* infections.

Anti-*Candida* activity of palmitic acid from *Streptomyces* sp. GMR22 is not explicitly detailed in the provided context. However, Nirwati et al. (2022) indicated that palmitic acid is a major constituent of the chloroform of GMR22, which possesses antifungal and antibiofilm properties against *C. albicans*. Their study also identified gene clusters related to the biosynthesis of palmitic acid, suggesting a metabolic pathway within *Streptomyces* sp. GMR22 that facilitated the production of this fatty acid [42]. While the mechanism of action is not directly addressed, it is known that fatty acids, such as palmitic acid, can disrupt fungal cell membranes, leading to cell lysis and death. This disruption is often due to the integration of fatty acids into the lipid bilayer, altering its integrity and fluidity [59]. Additionally, the antibiofilm activity may be attributed to the ability of palmitic acid to interfere with the adhesion and maturation processes of biofilm formation, although this is not explicitly stated in the context provided. In summary, while the specific mechanism of action of palmitic acid from *Streptomyces* sp. GMR22 against *C. albicans* was not detailed in the papers, it can be inferred that its antifungal properties may be related to its integration into fungal cell membranes and potential interference with biofilm formation. Further research would be required to elucidate the precise molecular interactions and pathways involved [42, 59].

Mechanism of action of the anti-*Candida* compounds Phenyl 2′α, 2′β, 6′β-trimethyl cyclohexyl ketone (1PB1) and Phenyl nonanyl ether (1PB2) isolated from *Streptomyces chrestomyceticus* strain ADP4 has been characterized through various studies. While the specific molecular interactions and pathways have not been fully elucidated, the available data suggest that these compounds exhibit their antifungal effects through different modes of action. Moreover, 1PB2 has been shown to be effective in inhibiting the biofilm formation of *Candida albicans*, which is a key virulence factor and a common cause of resistance to antifungal treatments [44]. The biofilm inhibition may involve disruption of the cell membrane and prevention of the yeast-to-hyphae transition, which is crucial for biofilm development [43]. Additionally, the metabolites of *S. chrestomyceticus* ADP4, including 1PB1 and 1PB2, have been reported to inhibit the production of secretory aspartic proteases, enzymes that contribute to the virulence of *C. albicans* by facilitating tissue invasion and immune evasion [43, 60]. In summary, the anti-*Candida* compounds 1PB1 and 1PB2 from *S. chrestomyceticus* ADP4 appear to act by inhibiting biofilm formation and possibly by interfering with the production of virulence factors, such as secretory aspartic proteases. These findings support the potential of these compounds as candidates for the development of new antifungal therapies, particularly against drug-resistant strains of *Candida* sp. However, further research is needed to fully understand the molecular mechanisms underlying their antifungal activity and to assess their efficacy and safety in clinical settings.

Actinomycin D, isolated from *Streptomyces luteus* and *Streptomyces parvulus*, exhibits antifungal activity against *Candida* species by damaging the fungal plasma membrane. Specifically, actinomycin D from *S. luteus* inhibits spore germination, hyphal growth, and biomass accumulation of *Verticillium dahliae*, a model for fungal pathogens, in a dose-dependent manner [61]. The mechanism involves a membrane-splitting action, as evidenced by flow cytometry, ergosterol measurement, cell leakage assays, and electron microscopy, which show swollen cells and cellular content leakage due to plasma membrane damage [61]. It is remarkable that while actinomycin D from *S. parvulus* has been shown to be effective against various bacteria, including *Staphylococcus aureus*, and to inhibit biofilm formation, its specific anti-*Candida* activity and mechanism of action are not detailed in the provided papers [62, 63]. However, the general antifungal mechanism of actinomycin D, as demonstrated by *S. luteus*, likely applies due to the similar structural properties of the compound. In summary, actinomycin D from both *Streptomyces* species functions as an antifungal agent primarily through disruption of the fungal plasma membrane, leading to cell damage and death. While the papers focus on different pathogens and aspects of the activity of actinomycin D, the underlying mechanism of membrane damage is a consistent theme in its antifungal action [61]. Further research is required to elucidate the specific effects and mechanisms of actinomycin D from *S. parvulus* against *Candida* species.

Mechanism of action of the anti-*Candida* compound 55B3 3-(dictyotin-11′-oate-15′α, 19′β-olide)-4-(dictyotin-11′-oate-15″α, 19″β-olide)-protocatechoic acid was not directly provided in the context of the provided papers. However, several studies have investigated the mechanisms of action of various antifungal compounds against *Candida* species, which may offer insights into potential mechanisms by which a compound, like 55B3, could operate. For instance, compounds have been shown to disrupt the fungal plasma membrane, inhibit ergosterol biosynthesis, and induce membrane depolarization and permeabilization [64–68]. It should be noted that while some compounds act by forming pores in the fungal membrane [65, 66], others have been found to inhibit specific pathways, such as the ergosterol biosynthesis pathway [68] or act as competitive inhibitors of enzymes, like acid trehalase [69]. Additionally, some studies have utilized model membranes to elucidate the antifungal mechanism, suggesting that compounds can induce leakage of intracellular components [66, 70]. In summary, while the specific mechanism of action for the compound 55B3 was not detailed in the provided papers, the general antifungal mechanisms described included disruption of the fungal plasma membrane, inhibition of ergosterol biosynthesis, membrane depolarization, and permeabilization, as well as pore formation. These mechanisms provide a framework within which the action of 55B3 might be understood, pending further specific research on this compound [64–68].

## Conclusion

This study confirmed the potential of *Streptomyces* compounds as *Candida* biofilm inhibitors. Several compounds showed high effectiveness, such as bafilomycin from *Streptomyces albolongus* with an MIC of 3.13 µg/ml against *Candida parapsilosis*, and palmitic acid from *Streptomyces* sp. GMR22 with an MIC of 50 µg/ml against *Candida albicans*. The phenyl nonanyl ether compound from *Streptomyces chrestomyceticus* was also effective with an MIC of 1.95 µg/ml against *Candida albicans*. These data show that *Streptomyces* compounds have great potential to be developed as new therapeutic agents against *Candida* biofilm infections. However, further research is needed to confirm their safety as well as their efficacy in clinical applications. The authors also suggest that future research focus on developing therapeutic strategies based on compounds produced by *Streptomyces*, especially compounds that show significant potential in inhibiting *Candida* biofilms. *In vivo* studies are needed to evaluate the safety and effectiveness of these compounds in complex clinical environments. In addition, further studies could explore the possibility of combined use of *Streptomyces* compounds with conventional antifungal agents to improve therapeutic efficacy, reduce antifungal resistance, and accelerate healing. Identification of more detailed molecular mechanisms of action is also important for a deeper understanding of the interaction of *Streptomyces* compounds with *Candida* biofilms, thereby enabling the design of more specific and effective drugs for clinical applications.
